# Molecular genetic analysis of spring wheat core collection using genetic diversity, population structure, and linkage disequilibrium

**DOI:** 10.1186/s12864-020-06835-0

**Published:** 2020-06-26

**Authors:** Amira M. I. Mourad, Vikas Belamkar, P. Stephen Baenziger

**Affiliations:** 1 Department of Agronomy, Faculty of Agricultural, Assuit University, Asyut, Egypt; 2 Department of Agronomy and Horticulture, Plant Science Hall, UNL, Lincoln, NE USA

**Keywords:** Linkage disequilibrium, Haplotype blocks, Genome-wide association study, Analysis of molecular variance, Genotype-by-sequencing

## Abstract

**Background:**

Wheat (*Triticum aestivium* L.) is an important crop globally which has a complex genome. To identify the parents with useful agronomic characteristics that could be used in the various breeding programs, it is very important to understand the genetic diversity among global wheat genotypes. Also, understanding the genetic diversity is useful in breeding studies such as marker-assisted selection (MAS), genome-wide association studies (GWAS), and genomic selection.

**Results:**

To understand the genetic diversity in wheat, a set of 103 spring wheat genotypes which represented five different continents were used. These genotypes were genotyped using 36,720 genotyping-by-sequencing derived SNPs (GBS-SNPs) which were well distributed across wheat chromosomes. The tested 103-wheat genotypes contained three different subpopulations based on population structure, principle coordinate, and kinship analyses. A significant variation was found within and among the subpopulations based on the AMOVA. Subpopulation 1 was found to be the more diverse subpopulation based on the different allelic patterns (*Na*, *Ne*, *I*, *h*, and *uh*). No high linkage disequilibrium was found between the 36,720 SNPs. However, based on the genomic level, D genome was found to have the highest LD compared with the two other genomes A and B. The ratio between the number of significant LD/number of non-significant LD suggested that chromosomes 2D, 5A, and 7B are the highest LD chromosomes in their genomes with a value of 0.08, 0.07, and 0.05, respectively. Based on the LD decay, the D genome was found to be the lowest genome with the highest number of haplotype blocks on chromosome 2D.

**Conclusion:**

The recent study concluded that the 103-spring wheat genotypes and their GBS-SNP markers are very appropriate for GWAS studies and QTL-mapping. The core collection comprises three different subpopulations. Genotypes in subpopulation 1 are the most diverse genotypes and could be used in future breeding programs if they have desired traits. The distribution of LD hotspots across the genome was investigated which provides useful information on the genomic regions that includes interesting genes.

## Background

Wheat (*Triticum aestivum* L.) is one of the most important cereal crops globally. It feeds more than a third of the human population around the world. The genome of bread wheat is an allohexaploid which contains three different genomes A, B, and D [1–3]. Generally, the genetic analysis of the wheat genome is very complex due to the polyploidy nature and the large genome size. The wheat genome is larger than *Arabidopsis thaliana* (~ 120 times), and *Oryza sativa* L. (~ 40 times) [4–6]. To well understand the complexity of the wheat genome, it is required to use good type of molecular markers which reduces the size of this genome by digesting it to multiple parts using restriction enzymes.

Generally, there are many types of molecular markers which could be used in various genetic analysis such as genetic diversity, genome-wide association studies, fingerprinting, evolutionary origin, and breeding applications. The most common type of markers is single nucleotide polymorphisms (SNPs) and simple sequence repeats (SSRs) [7]. However, by comparing SNPs and SSR markers, it was found that SNPs are excellent markers for studies that require a high number of markers such as association studies, QTL mapping, population structure, and genomic selection [8–12]. Recently, new techniques of sequencing have been developed to produce high-density genome-wide markers. Genotyping-by-sequencing (GBS) is one of these techniques which uses two different types of restriction enzymes (*PstI/MspI*) to reduce the complexity of large genomes such as wheat [13, 14]. Using the GBS technique provides many advantages such as; low cost, fewer purification steps, and easy sample handling [15].

Understanding the linkage disequilibrium (LD) between marker pairs is very important in association mapping studies as it determines the resolution of the association [16]. For example, if the LD rapidly decays, the resolution of the association will be high and vice versa [17]. Many previous studies discussed the relationship between LD decay and the resolution of association mapping in the wheat genome using different kinds of markers such as SSR and DArT and found that the LD varied among different wheat populations [18–21]. To achieve a high-resolution association mapping, a large number of markers should be used. GBS method produces such a high number of markers distributed across the genome.

As wheat is one of the most important crops globally, it is very important to study the global genetic variation. This requires the collection of cultivars from different countries. The USDA-ARS national plant germplasm system is a good resource for plant breeders worldwide as it contains a large number of accessions of wheat (~ 58,000) which have been collected starting from 1897. In 1995, the number of NSGC core accessions has been reduced to only 10% of the total number of the collected accessions following Brown 1989 [22] outline as described in Bonman et.al [23].. Following this outline, a collection of wheat accessions from all countries has resulted. This core collection, or a sample from it, could be considered as an ideal collection to study the genetic diversity of worldwide wheat germplasm. Consequently, understanding the genetic diversity in wheat germplasm is critical in breeding programs as it enables the wheat breeders to select the appropriate parents for the different breeding purposes. It is also very important in further breeding studies such as marker-assisted selection (MAS), genome-wide association studies (GWAS), and genomic selection. In the current study 103 spring genotypes representing 14 countries were collected from USDA gene bank and tested for their agronomic traits under the Egyptian conditions to increase the genetic diversity of adapted wheat genotypes in Egypt.

The objectives from this study were to (1) understand the genetic diversity and population structure in spring wheat using 103-accessions representing different countries worldwide, (2) compare the genetic properties among subpopulations, and (3) determine the patterns of linkage disequilibrium (LD).

## Results

### Distribution of SNP markers across the different wheat genomes

**Fig. 1:**
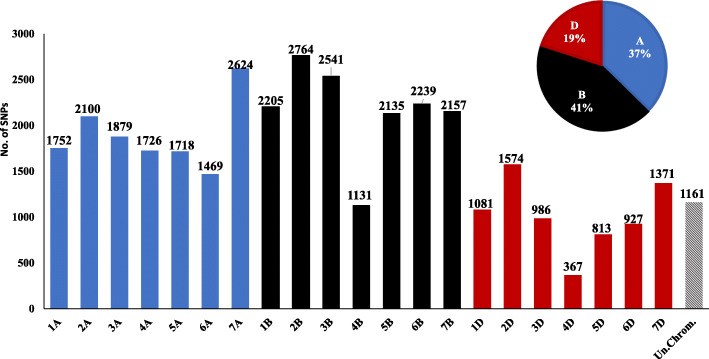
The distribution of the 36,720 SNPs across the 21 chromosomes in the 103-spring wheat panel

The total number of GBS derived SNPs from the tested genotypes was 287,798 SNPs. After quality filtering, the total number of high-quality SNPs was 36,720 which were well distributed across the genome (Fig. 1). The highest number of SNPs was located on genome B with a percentage of 41% (15,172 SNPs) while, the lowest number of SNPs located on genome D with a percentage of 19% (7119 SNPs). There were 1161 SNPs located within scaffolds with an unknown chromosomal location. The number of SNPs/chromosome (Chro.) ranged from 367 SNPs (4D Chro.) to 2764 SNPs (2B Chro.).

### Genetic diversity and the polymorphism information content (PIC)

**Fig. 2:**
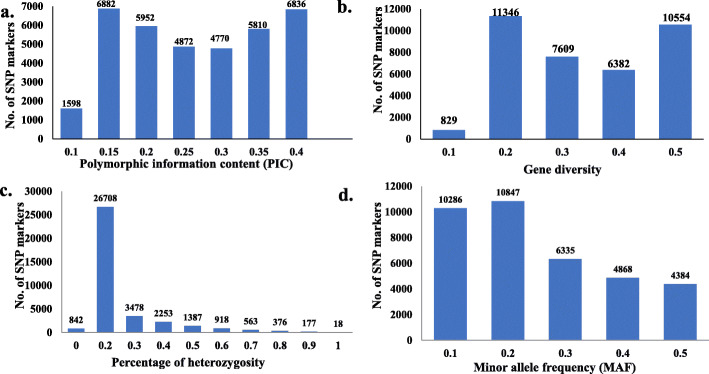
The distribution of polymorphic information content (PIC) (**a**), gene diversity (**b**), percentage of heterozygosity (**c**), and minor allele frequency (**d**) for the 37,295 SNP markers in the 103-spring wheat panel

The PIC value across chromosomes ranged from 0.1 (1598 SNPs) to 0.4 (6836 SNPs) with an average of 0.24 (Fig. 2a). Gene diversity (GD) ranged from 0.1 (829 SNPs) to 0.5 (10,554 SNPs) with an average of 0.29. The percentage of heterozygosity extended from 0% (842 SNPs) to 100% (18 SNPs) with an average of 0.15, respectively (Fig. 2b and c). Minor allele frequency ranged from 0.1 (10,286 SNPs) to 0.5 (4384 SNPs) with an average of 0.21 (Fig. 2d).

### Population structure and relationships

**Fig. 3:**
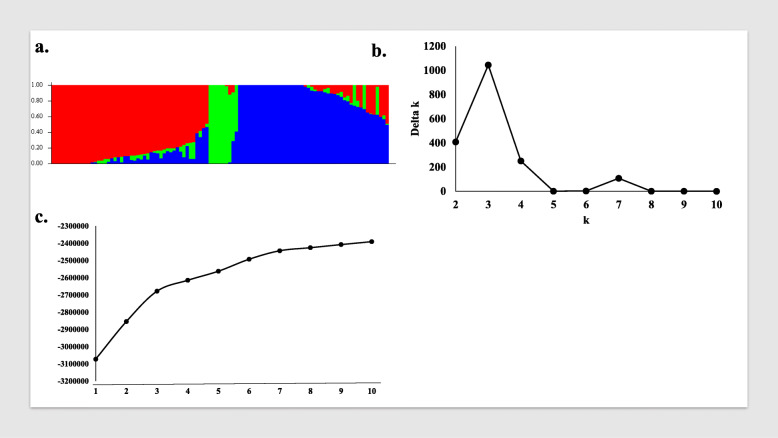
Analysis of population structure using 36,720 SNP markers: (**a**) estimated population structure of 103-spring wheat genotypes (k = 3). The y-axis is the sub-population membership, and the x-axis is the genotypes, and (**b**) delta (Δ) K for different numbers of sub-populations, and (**c**) the average of log-likelihood value

**Fig. 4:**
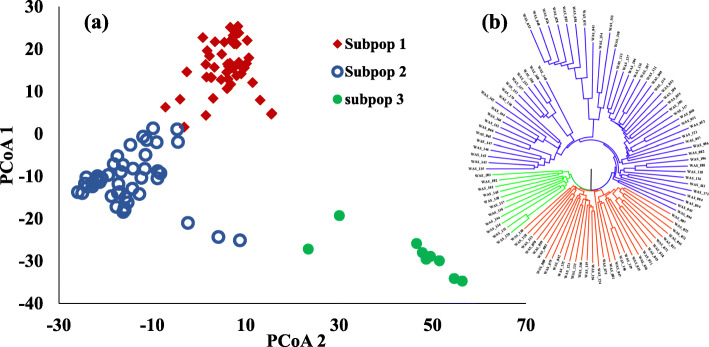
**a** Principle coordinates analysis (PCoA) based on genetic distance (SNPs), **b** Dendrogram analysis based on the genetic distance calculated by UPGMA

The STRUCTURE analysis software was used to identify the number of subpopulations in the tested 103 genotypes (Fig. 3). The number of clusters (*K*) was plotted against Δ*K* to identify the suitable number of subpopulations. The largest Δ*K* value was observed at *K* = 3 suggesting the presence of three subpopulations in the tested genotypes (Fig. 3a and b). As illustrated in Fig. 3c, there is a continuous-gradual increase in the assessed log-likelihood with the increase in the number of *K* confirming the presence of three subpopulations in the tested genotypes with the highest probability. The three groups consist of 48, 46, and nine genotypes for the red, blue, and green group, respectively (Fig. 3 and Table 1). By comparing the results of STRUCTURE software and the principle coordinate analysis, we found that both are in agreement and dividing the tested genotypes into three groups (Fig. 4 a and b). Based on both analyses, the first group (48 genotypes) contained all of the genotypes from Australia, Germany, Greece, and Kenya while, the second subpopulation (46 genotypes) contained the genotypes from Algeria, Ethiopia, and Tunisia. The genotypes from the remaining countries such as Egypt, Afghanistan, Canada, Iran, Kazakhstan, Morocco, Saudi Arabia, and Oman were distributed among the three groups. For example, most of the Egyptian genotypes belonged to the first group except for six genotypes that belonged to the third group. The percentage of the membership of each country in the three subpopulations is presented in Table 2.

**Table 1:** STRUCTURE analysis of 103-spring wheat genotypes for the fixation index (Fst) (significant divergences), average distance (expected heterozygosity) and number of genotypes in each subpopulation

Subpopulation	Fst ^**a**^	Exp. Hetero ^**b**^	No of genotypes
**Subpopulation 1**	0.1984	0.2671	48
**Subpopulation 2**	0.6142	0.1776	46
**Subpopulation 3**	0.3090	0.2325	9

^a^Fst is a measure of genetic differentiation; ^b^Expected heterozygosity

**Table 2:** The percentage of the membership of each country in the three subpopulations

Country	Subpopulation 1	Subpopulation 2	Subpopulation 3	Number of genotypes
**Afghanistan**	11.11	88.89	0.00	9
**Algeria**	0.00	100.00	0.00	1
**Australia**	100.00	0.00	0.00	1
**Canada**	80.00	20.00	0.00	5
**Egypt**	64.71	0.00	35.29	17
**Ethiopia**	0.00	100.00	0.00	1
**Germany**	100.00	0.00	0.00	2
**Greece**	100.00	0.00	0.00	3
**Iran**	7.14	92.86	0.00	14
**Kazakhstan**	75.00	25.00	0.00	8
**Kenya**	100.00	0.00	0.00	5
**Morocco**	64.29	35.71	0.00	14
**Oman**	0.00	87.50	12.50	8
**Saudi Arabia**	28.57	71.43	0.00	7
**Tunisia**	0.00	100	0.00	1
**Unknown countries**	42.86	28.57	28.57	7

**Fig. 5:**
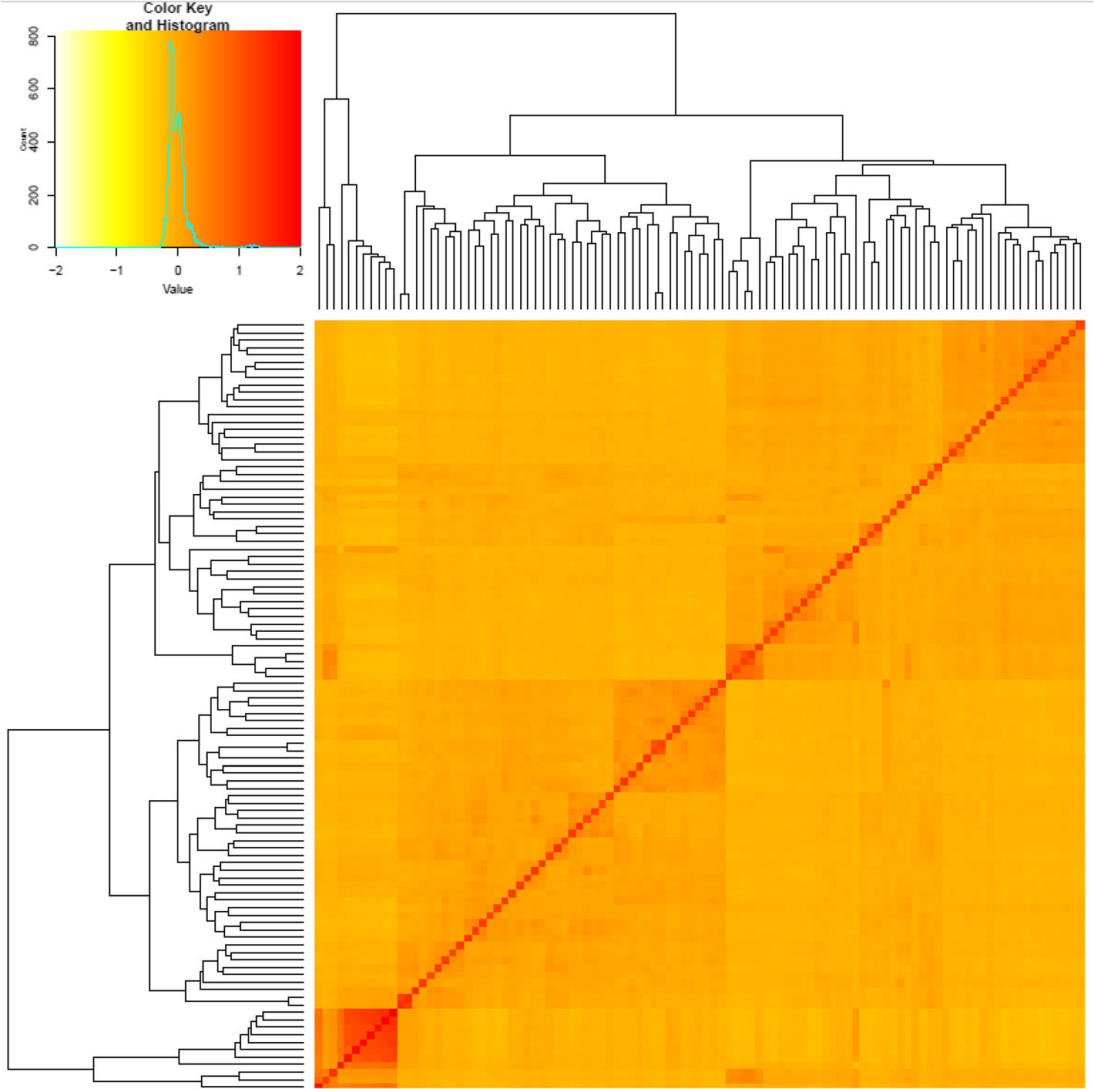
Heat map of kinship matrix with the dendogram shown on the top and left based on the 36,720 SNP markers

Significant genetic differentiation was found among the three subpopulations and expected heterozygosity (average distance) among genotypes in each subpopulation (Table 1). Subpopulation 1 had the highest value of expected heterozygosity with a value of 0.2671, followed by the third subpopulation (0.23526) and the second subpopulation (0.1776). The Fixation index (Fst) could be considered as the best index for the determination of the overall genetic variation among subpopulations. In our studied materials, the highest genetic variation was found in subpopulation 2 with the Fst value of 0.6142. While subpopulation 1 showed lower genetic variation among its genotypes with the Fst value of 0.1984 (Table 1). The analysis of kinship is illustrated as a genetic clustering and indicated that the current panel of genotypes was divided into three possible subgroups, with considerable genetic differences among the genotypes (Fig. 5).

### Genetic differentiation of populations

The three subpopulations identified based on STRUCTURE analysis were used to calculate the AMOVA and genetic diversity indices in GenAlex 6.41 software. A significant variation within and among the subpopulations was found based on the AMOVA results. The total variation between the tested genotypes could be classified into two parts; variation among subpopulations with a percentage of 15%, and variation within subpopulations with a percentage of 85% (Table 3). The haploid number of migrants (Nm) was 2.90 indicating that there is a high gene exchange among subpopulations.

**Table 3:** Analysis of molecular variance using 36,720 SNPs and the genetic differentiation among the three subpopulations of the 103-spring wheat panel

Source	df	SS	MS	Est. Var.	%	***P*** value
**Among Pops**	2	47,935.156	23,967.578	676.092	15	0.001
**Within pops**	100	392,111.058	3921.111	3921.111	85	0.001
**Total**	102	440,046.214		4597.203	100	0.001
**Nm (haploid)**	2.900					

### The allelic pattern across the populations

The average number of different alleles (Na) and effective alleles (Ne) were 2.528 and 1.781, respectively (Table 4). The Shannon index (*I*), the diversity index (*h*), and the unbiased diversity index (*uh*) had average values of 0.636, 0.384, and 0.403 based on the average of the three subpopulations (Table 4). Based on all allelic patterns, subpopulation 1 was the most diverse subpopulation when compared to subpopulations 2 and 3 as it has higher numbers of all the diversity indices. Subpopulation 3 was the least diverse subpopulation based on all indices as might be expected with its low number of lines. The percentage of polymorphic loci within subpopulations was 99.71, 99.39, and 64.84 for the first, second, and third subpopulation, respectively with an average of 87.99%.

**Table 4:** Mean of different genetic parameters including number of different alleles (*Na*), number of effective allele (*Ne*), Shannon’s index (*l*), diversity index (*h*), unbiased diversity index (*uh*), and percentage of polymorphic loci (*PPL*) in each subpopulation of the 103-genotypes

Subpopulations	*Na*	*Ne*	*I*	*h*	*uh*	*PPL*
**Subpopulation 1**	2.897	1.994	0.782	0.471	0.482	99.71
**Subpopulation 2**	2.869	1.921	0.002	0.445	0.457	99.39
**Subpopulation 3**	1.816	1.429	0.380	0.236	0.271	64.87
**Mean**	2.528	1.781	0.636	0.384	0.403	87.99

### Evaluation of linkage disequilibrium

The analysis of linkage disequilibrium showed that the LD decayed with the genetic distance (Supplementary Fig. 1). The values of *R*^2^ revealed that there is no high LD among the 36,720 SNP pairs in the tested genotypes with an average value of 0.138 (Table 5). However, it was more useful to test the LD between each pair of SNPs located on the same chromosome and determine the average of the LD in each genome to identify the pattern of LD in the three genomes. Table 5 represents the average LD/chromosome and the number of significant and nonsignificant LD between each pair of SNPs located on the same chromosome. At the genome level, the highest LD was found in the D genome with an average of 0.1853, while the LD on both A and B genomes was almost the same with an average of 0.1189 and 0.1124, respectively. The LD within each genome ranged from 0.106 (1A) to 0.125 (4A), 0.098 (6B) to 0.122 (4B) and 0.167 (4D) to 0.241 (2D). The significance of LD between each SNP pair located on the same chromosome was tested using Bonferroni correction (α = 0.01). The D Genome contained the highest significant LD based on the average of chromosomes with *R*^2^ = 0.887 followed by genomes A and B with an average *R*^2^ of 0.818 and 0.815, respectively. Likewise, the highest LD as an average of all SNP pairs with non-significant LD was found in genome D (0.149), while the LD average of non-significant markers was approximately the same in genome A and B with an average of ~ 0.084.

**Table 5:** Linkage disequilibrium between SNP markers located on the same chromosome and genome

Chromosome	R^2	Number sig. LD	Average Sig. LD	Percentage of sig. R^2	Number non sig. LD	Average non sig. LD	No. of sig. LD/ No. of non sig. LD
1A	0.106696275	2673	0.773570652	4.6	55,965	0.074845024	0.05
2A	0.117889775	2973	0.79594235	4.8	58,919	0.083675849	0.05
3A	0.112887651	1876	0.852327861	3.4	54,032	0.087214164	0.03
4A	0.125257515	2590	0.862161693	4.6	53,148	0.089346816	0.05
5A	0.125428444	3419	0.809304824	6.2	52,153	0.080595484	0.07
6A	0.120074851	2829	0.794986633	5.9	44,846	0.077499696	0.06
7A	0.124468994	3482	0.835755958	3.9	86,668	0.095892112	0.04
mean	0.118957644	19,842	0.817721425	4.7	405,731	0.084152735	0.05
1B	0.114425037	2767	0.804864224	3.9	68,196	0.086411	0.04
2B	0.108414675	2979	0.821397105	3.4	85,494	0.083571122	0.03
3B	0.115633024	3343	0.797272582	4.0	80,596	0.087359648	0.04
4B	0.122410098	1520	0.837638581	4.0	36,103	0.092297717	0.04
5B	0.106133555	3151	0.828076529	4.2	72,350	0.074692834	0.04
6B	0.098446778	2543	0.799670654	3.4	72,669	0.073907947	0.03
7B	0.121483303	3397	0.814441598	4.8	67,784	0.086755649	0.05
mean	0.112420924	19,700	0.814765896	3.9	483,192	0.083570845	0.04
1D	0.186308632	1559	0.859202458	6.3	23,371	0.141422172	0.07
2D	0.240878007	2986	0.929159518	7.7	36,041	0.183853824	0.08
3D	0.206075532	1320	0.901496541	6.4	19,422	0.158811824	0.07
4D	0.16616349	239	0.905766016	3.7	6244	0.137853911	0.04
5D	0.178633759	505	0.89830723	3.6	13,699	0.152103713	0.04
6D	0.145398046	606	0.818621715	3.0	19,755	0.124746388	0.03
7D	0.173585725	1134	0.893450395	3.7	29,693	0.146093503	0.04
mean	0.185291884	8349	0.886571982	5.3	148,225	0.149269334	0.06
Genome mean	0.137984						

**Fig. 6:**
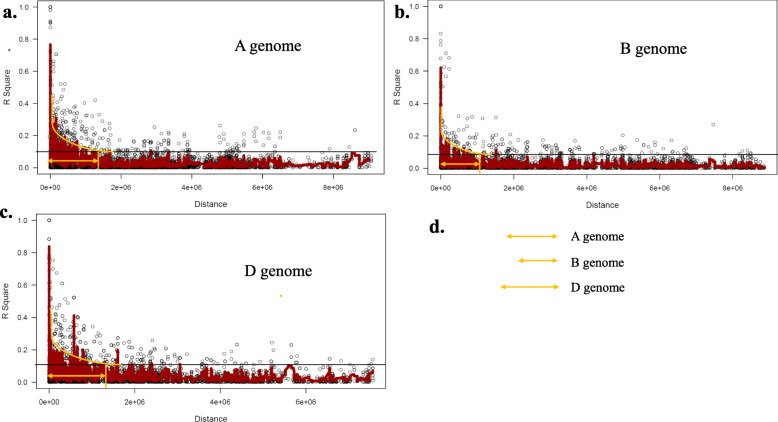
The rate of linkage disequilibrium (LD) decay of the genome A (**a**.), genome B (**b**.) and genome D (**c**.) of the 103-spring wheat based on the 36,720 SNP markers. (**d**.) comparison between LD decay (distance) among the three genomes

The ratio between the number of significant LD and the number of nonsignificant LD could be arranged from higher to lower as follows; 0.06, 0.05, and 0.04 for genome D, genome A, and genome B respectively. At the chromosome level, chromosomes 2D, 5A, and 7B had the highest ratios between the number of significant and non-significant LD with values of 0.08, 0.07, and 0.05, respectively. The R^2^ between each pair of markers was plotted against genetic distance (kb). The LD decay in each genome is illustrated in Fig. 6 and whole-genome in Supplementary Figure 1. The LD decay in the D genome was slower than the LD decay in A and B genomes. The LD decay in A genome was slower than the B genome (Fig. 6a-d). The number of haplotype blocks was investigated for the highest three chromosomes. Chromosome 2D was found to contain 28 haplotype blocks followed by chromosome 5A and 7B which contain 12 and 11 blocks, respectively (Supplementary figure 2).

## Discussion

The studied wheat genotypes were collected from different countries representing five of the world continents (Africa, Europe, Asia, North America, and Australia) which enable us to estimate wheat genetic diversity in the studied countries. The study was conducted using 36,720 SNPs which were well distributed across the three hexaploid wheat genomes (A, B, and D). The highest number of SNPs were found on genome B (41%), while the lowest number of SNPs were found on genome D (19%) indicating that genome D is the least diverse wheat genome (Fig. 1). The D genome was reported to be the least diverse genome in previous studies which used different types of markers such as GBS-SNPs, RFLP, SSR, AFLP, and DArT markers [24–30]. Dubcovsky and Dvorak [1] concluded that the proportion of diversity in *Triticum aestivum* L. resulted in the polyploid nature of its tetraploid ancestor with AABB. This conclusion could be a good explanation of the high level of diversity among hexaploid wheat genotypes and the high number of SNPs in the A and B genomes.

The PIC values and genetic diversity are very helpful parameters to measure the polymorphism between the genotypes used in breeding programs. Generally, for multi-locus markers such as SSR markers, the PIC values range from 0 to 1.0. According to Botstein et.al [31, 32], multi-allelic markers could be classified into three categories based on their PIC values. These three categories are: (1) highly informative markers with PIC values higher than 0.5, (2) moderately informative marker with PIC value ranging from 0.25 to 0.5, and (3) slightly informative markers with PIC values less than 0.25. However, for the bi-allelic markers like SNPs, the highest PIC value is 0.5. As a result of this bi-allelic nature, SNP markers could be considered as moderate to low informative markers. The average PIC value obtained in this study is 0.24 which is similar to PIC values in previous studies [24, 33]. This PIC value was reported as a good indicator of informative markers which could be used in studying the genetic diversity in the different organisms [34]. Based on the PIC values in our tested population and the good distribution of the studied SNP markers, we can conclude that these markers explained the genetic diversity in spring wheat and could be used in other genetic studies such as genome-wide association study to identify alleles controlling target traits.

### Population structure and relationships

Studying the population structure is very helpful in understanding the genetic diversity of the tested genotypes. This is the first step in conducting the association mapping studies. In our tested materials, STRUCTURE analysis, as well as the PCoA, confirmed the presence of three subpopulations. In each subpopulation, there were genotypes from different countries and continents. This result was expected due to the continuous gene flow of wheat genotypes among the different countries historically to the present. This exchange resulted in the presence of diverse genetic backgrounds in the same country, and thus the presence of genotypes from different countries in the same subpopulation. The majority of genotypes from Afghanistan, Iran, Oman, and Saudi Arabia were clustered together in subpopulation 2, while, the majority of genotypes from Egypt, Canada, Kazakhstan, and Morocco were grouped in subpopulation 1. Genotypes from Germany, Greece, and Kenya were grouped in only one subpopulation. This information is very important in selecting the candidate parents for target traits in breeding programs as genetic distance should be highly considered. Genetic diversity between two genotypes from two different countries representing two different continents may be very low and not useful to use such parents in breeding programs. Understanding this presence of population structure in the tested 103-spring wheat genotypes is very important. It must be taken into account before conducting genome-wide association studies (GWAS) as it could result in a superior association between the studied trait and the GBS-derived SNPs [35].

### Genetic differentiation of populations

The result of AMOVA indicated the presence of highly significant genetic diversity among the three subpopulations (Table 2). The high level of genetic diversity within the subpopulations could be due to the selection for specific traits that have been done by the wheat breeders in the different countries for specifically targeted traits. In addition, each subpopulation had wheat genotypes from different countries. While the low level of genetic diversity among the populations (15%) could be due to gene flow resulted from the wheat germplasm flow among the different regions. Therefore, it could be more useful to select genotypes as parents, in the breeding programs for improving target traits, from the same subpopulation than selecting from different subpopulations. However, this may change depending on the breeding goals. Making crosses among genotypes from different subpopulations may be required to incorporate haplotypes from different founder populations. Similar results of high genetic diversity within the subpopulations and low diversity among the subpopulations were found in winter and synthetic wheat genotypes [24, 36]. In order to identify the level of gene flow among the subpopulation, Nm (haploid) was calculated. It was reported that, if the Nm (haploid) value was 1.00 or lower, this indicates the low level of gene flow [37]. In our tested materials, Nm (haploid) was 2.900 which is much higher than 1.00 indicating the high level of gene flow between the subpopulations. This result supports the distribution of the genotypes from one country in the three subpopulations in the tested plant material.

Based on all the allelic pattern indices (*Na*, *Ne*, *I*, *h*, *uh,* and *PPL*) among the three subpopulations, subpopulation 1 is the most diverse subpopulation as it shows the highest values of all the indices. This result is expected as this subpopulation contains genotypes from 11 different countries compared with the other two subpopulations which contain genotypes from ten and two different countries, respectively (Supplementary Table 1). Based on these results, we can conclude that the studied 103-spring wheat genotypes, especially subpopulation 1, provide a broad and useful source of genetic diversity in wheat. This set of genotypes could be used in future breeding programs to increase the genetic diversity among wheat genotypes. Increasing genetic diversity is very useful in conducting genome-wide association studies (GWAS) and marker-assisted selection (MAS) for identifying genes controlling important traits in wheat. Moreover, selection among the genotypes in subpopulation 1 for target traits will be fruitful for the genetic improvement of wheat.

### Linkage disequilibrium and kinship between the studied genotypes

The determination of the LD magnitude and decay is very important as they affect the resolution of association mapping and the SNP markers required for conducting association studies [16]. The extent of LD differs across genomes in many species. As wheat has three different genomes, we analyzed the LD decay in each genome. The LD decay was estimated when the values of LD declined below 0.1 based on the curve of the nonlinear logarithmic trend line. The LD decayed in genome D at higher distances than genomes A and B. The lowest rate of LD decay was observed in genome B. This result suggested that fewer markers are needed to detect target QTLs located on genome D using GWAS than those needed for detecting QTLs on the other genomes [38]. By looking at the number of markers in each genome, the D genome had the lowest number of SNPs (20%) followed by genomes A and B, respectively. This indicates that the current set of material and SNPs are very appropriate to conduct GWAS to identify alleles associated with target traits in wheat. The high and low LD found across the three genomes provide a high chance to detect target QTL with large and small effects in the current materials [39]. The same results of LD decay pattern across the three genomes of wheat were reported by Liu et al. (2017) and Ayana et al. (2018) [38, 40].

Interestingly, high LD regions at a high genetic distance were observed in each genome. These high LD regions which were among low LD regions are called LD hotspots. Visibly, LD hotspot regions in genome A and D were higher than those in genome B. According to the LD significance level between the markers, the ratio of the number of significant to non-significant markers was higher in genome D (6%) and A (5%) than in genome B (4%) which means that genome B had the highest number of markers in non-significant LD (Table 5). Therefore, it is very important to understand the structure of LD in the wheat population and the distribution of LD hotspot regions in each genome. Understanding the LD structure enables to identify the genetic regions associated with agronomic traits and determining the density of markers needed to associate the genotypes with the studied traits [16].

The pattern and number of LD hotspots in the genome provide useful information in determining marker density. The greater the recombination rate, the greater the need for higher marker density as the greater chance for the LD to be broken by a recombination event when QTL and the marker are close together [41]. By looking at the LD plot including the three genomes (supplementary Figure 2), hotspots genomic regions were clearly found at a high genetic distance and separated the low LD regions.

## Conclusion

In conclusion, the analysis of population structure and LD decay were genetically dissected in a set of 103 wheat core collection genotypes from different countries. The current material was divided into three possible subpopulations. The most diverse genotypes were found in subpopulation 1 and they can be used in the future breeding program by crossing among parents with target traits. The population structure was also very useful to determine the appropriate GWAS statistical methods that can be used to detect QTLs in these populations. Moreover, the genetic diversity of markers in the current population suggested that the markers are informative and polymorphic. The genetic properties of this population including the number of genotypes and SNP markers allow this population to be used for further genetic studies to genetically improve spring wheat through advanced breeding programs. We identified the distribution of LD hotspots across each genome and the whole genome which provided useful information on the possibility of genomic regions that may include interesting genes.

## Methods

### Plant materials

A set of 103 spring bread wheat genotypes were obtained from the USDA-ARS worldwide core collection and used in this study. The genotypes are representing fourteen different countries (Table 2). Out of the 103 tested genotypes, fifteen are local varieties that are usually planted in Egypt. The remaining 88 genotypes were from other countries, evaluated in Egypt, and found to perform well. Hence those 88 genotypes were adapted to the Egyptian environmental conditions and would be used as global parents for cultivar improvement.

### DNA extraction, genotype-by-sequencing (GBS), and SNP calling

DNA was extracted from all the tested genotypes from 2 to 3 leaves of 2 weeks old seedlings using BioSprint 96 DNA Plant Kits (Qiagen, Hombrechtikon, Switzerland). The extracted DNA was digested for GBS purpose using two different restriction enzymes, *PstI* and *MspI.* The Illumina, Inc. NGS platforms were used to generate the sequencing of the pooled libraries. TASSEL 5.0 v2 software GBS pipeline was used to identify SNPs [42]. Chinese Spring genome v1.0 from the International Wheat Genome Sequencing Consortium (IWGSC) was used as a reference genome for SNP calling and GBS tags were aligned using Burrows-Wheeler Aligner [43]. Generated SNPs were filtered for minor allele frequency (MAF) less than 5%, missing data less than 20%, missing genotypes less than 30%, and maximum heterozygosity 35%.

### Data analysis

#### Genetic properties of markers

Genetic diversity statistics of all the 36,720 SNP markers such as polymorphic information content (PIC), gene diversity, percentage of heterozygosity, and minor allele frequency (MAF) were calculated using PowerMarker software V 3.25 [44]. The following formula was used to calculate the PIC according to [31].

$$ PIC=1-{\sum}_{j=1}^n{P}_{ij}^2-{\sum}_{j=1}^{n=1}{\sum}_{k=j+1}^n2{P}_{ij}^2{P}_{ik}^2 $$

Where P_ij_ and P_ik_ are the frequencies of j_th_ and k_th_ alleles for marker i, respectively.

#### Analysis of population structure

To estimate the number of subpopulations in the current tested genotypes, a model-based (Bayesian) method with the filtered SNPs (36,720) was used. STRUCTURE 3.4.0 software was used to analyze population structure [45]. Burn-in iteration was 100,000 followed by 100,00 Markov chain Monte Carlo (MCMC) replications after burn-in for each run. In this analysis, allele frequencies and the admixture correlated models were considered. Five impended iterations were used in the STRUCTURE. The hypothetical number of the subpopulation (k) extended from 1 to 10. STRUCTURE HARVESTER [46] was used to identify the best k, where k is the number of subpopulations [47]. The genetic distance among the tested genotypes was calculated using TASSEL v.5.2.5 software [42]. Based on this genetic distance, principal coordinate analysis (PCoA) was performed.

#### Analysis of molecular variance (AMOVA) and genetic diversity indices

For this analysis, 14,400 SNPs based on the highest PIC values (from 0.282 to 0.375) were used. The number of subpopulations based on the STRUCTURE analysis was considered in the AMOVA. The genetic indices such as fixation index (F_st_), different alleles (*Na*), number of effective alleles (*Ne*), Shannon’s index (*I*), the diversity index (*h*), the unbiased diversity index (*uh*), and percentage of polymorphic loci (*PPL*) were calculated. The AMOVA and estimation of genetic indices were performed using GenAlex 6.41 [48].

#### Linkage disequilibrium (LD) structure

The LD between each pair of the 36,720 SNPs was calculated as the squared allele frequency correlation coefficient (*R*^2^) using TASSEL v.5.2.5 software [42]. The LD was calculated separately for each chromosome in each genome (A, B, and D) in order to understand the structure of LD in the current population. To identify the significant LD, Bonferroni correction (α = 0.01) was applied [12]. The kinship matrix between the tested genotypes as well as the LD decay for each genome was calculated using GAPIT, R package [49].

### Haplotype block analysis

In each genome, the chromosome contains the highest significant LD percentage was tested for the number of haplotype blocks using Haploview 4.2 software [50]. To perform this, the SNP data for the target chromosome was used to calculate the pair-wise LD between SNPs. The haplotype blocks were constructed using the four-gamete method and a cutoff 1% was used [51, 52].

## Supplementary information

**Additional file 1: Table S1.** List of genotypes in each subpopulation and their country.

**Additional file 2: Figure S1.** The rate of linkage disequilibrium (LD) decay of the 103-spring wheat based on the 36,720 SNP markers.

**Additional file 3: Figure S2.** Haplotype block analysis represents the number of haplotype blocks on: (a) chromosome 2D, (b) chromosome 5A, and chromosome 7B.

## Data Availability

Sequence data is available with the authors. The datasets used and analyzed during the current study are available from the corresponding author on reasonable request.

## Abbreviations

GBS: Genotyping-by-sequencing
GWAS: Genome-wide association study
MAS: Marker-assisted selection
LD: Linkage disequilibrium
