# Supplementary material for: Molecular genetic analysis of spring wheat core collection using genetic diversity, population structure, and linkage disequilibrium
Source: BMC Genomics. 2020 Jun 26;21:434. doi: 10.1186/s12864-020-06835-0 (PMC7318758; doi:10.1186/s12864-020-06835-0)
Supplement: Supplementary file 2 — Additional file 2: Figure S1. The rate of linkage disequilibrium (LD) decay of the 103-spring wheat based on the 36,720 SNP markers. [file 12864_2020_6835_MOESM2_ESM.pdf]

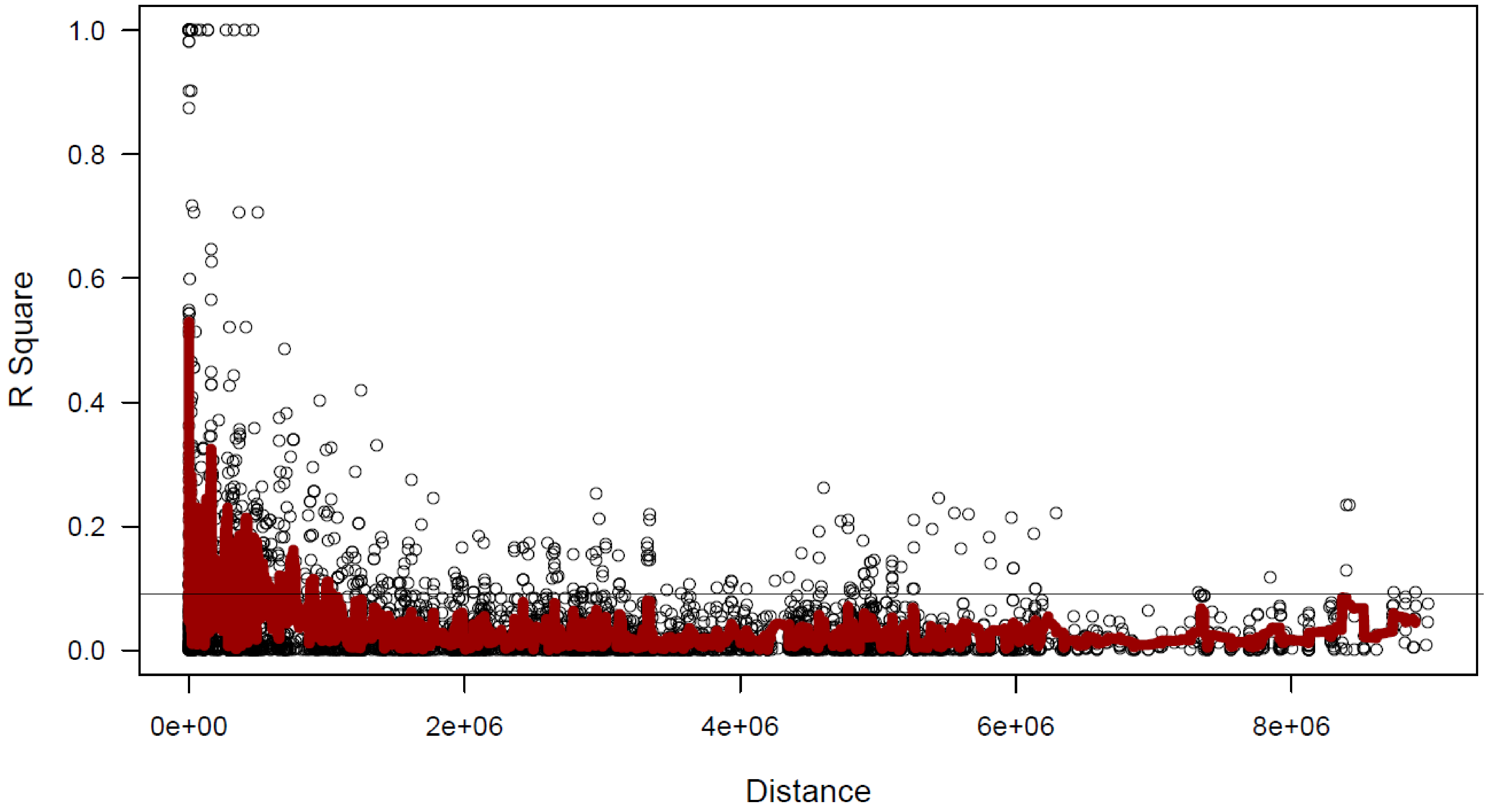

Supplementary Figure 1. The rate of linkage disequilibrium (LD) decay of the 103 spring wheat based on the 37295 SNP markers.
