# Supplementary material for: Molecular genetic analysis of spring wheat core collection using genetic diversity, population structure, and linkage disequilibrium
Source: BMC Genomics. 2020 Jun 26;21:434. doi: 10.1186/s12864-020-06835-0 (PMC7318758; doi:10.1186/s12864-020-06835-0)
Supplement: Supplementary file 3 — Additional file 3: Figure S2. Haplotype block analysis represents the number of haplotype blocks on: (a) chromosome 2D, (b) chromosome 5A, and chromosome 7B. [file 12864_2020_6835_MOESM3_ESM.pdf]

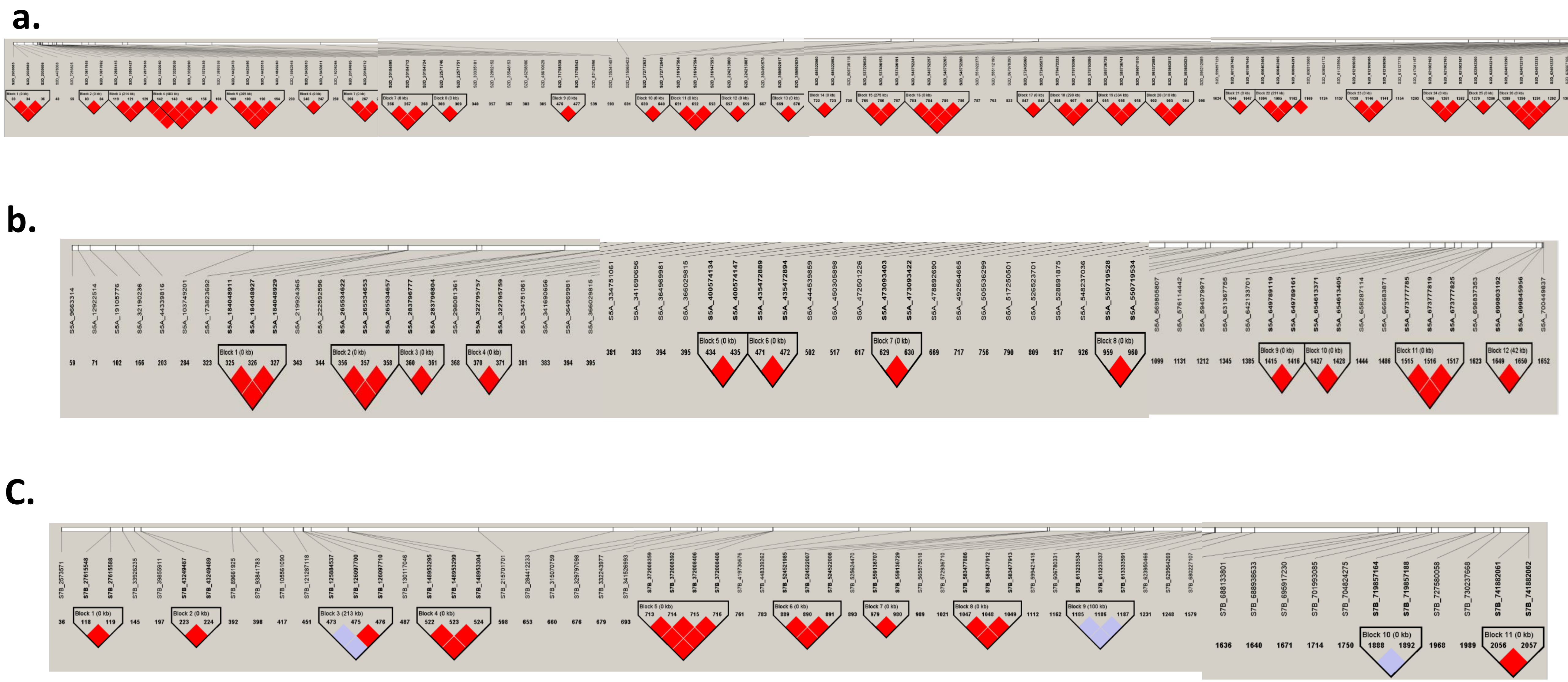

Supplementary Figure 2. Haplotype block analysis represents the number of haplotype blocks on: (a) chromosome 2D, (b) chromosome 5A, and chromosome 7B.
